# Paired assessment of liver telomere lengths in hepatocellular cancer is a reliable predictor of disease persistence

**DOI:** 10.1042/BSR20160621

**Published:** 2017-03-15

**Authors:** Wendu Feng, Decai Yu, Binghua Li, Ou-yang Luo, Tiancheng Xu, Yajuan Cao, Yitao Ding

**Affiliations:** 1 Department of Hepatobiliary Surgery, The Affiliated Drum Tower Hospital, Medical School of Nanjing University, Nanjing, Jiangsu 210093, China; 2 Jiangsu Key Laboratory of Molecular Medicine, Medical School of Nanjing University, Nanjing, Jiangsu 210093, China

**Keywords:** Biomarker, telomere, HCC, HBV, HCV

## Abstract

In the present study, we used a small series of highly defined patients, where we had matched timed peripheral blood samples (PBS), as well as paired liver biopsies obtained during collection of blood samples from patients with diagnosed hepatocellular carcinoma (HCC) and compared the correlation between the changes of telomere lengths in these defined samples. Patients included had either HCC alone or in conjunction with either pre-existing hepatitis B virus (HBV) or hepatitis C virus (HCV) infection. PCR-based assay incorporating primers to the telomeric hexamer repeats to polymerize and detect telomeric DNA was used. The average telomere length for each independent assessment was measured by seeing the differences in the intensity of the sample’s telomere signal (T) to the signal from a single-copy gene (S-, β-globin) to estimate the standard ratio. Our results provide the first convincing evidence that PBS may be utilized to assay telomere shortening as a predictor for disease persistence in HCC resulting after HBV or HCV infection, but not in non-infectious cause-stimulated HCC. These findings provide incipient opportunity to develop telomere length assessment as a biomarker tool for prediction of HCC in patients with HBV or HCV infection, as well as to gauge responses to chemotherapy and other treatment modalities.

## Introduction

Telomeres are specialized nucleoprotein repeat sequences that polarize the ends of linear chromosomes [1,2]. Telomeres are constituted by double-stranded TTAGGG DNA repeat hexamer sequences and a set of associated shelter-in complex proteins. Telomere is a critical genomic component for maintaining the overall stability and chromosomal intactness by preventing stoppage of premature replication. Telomeres are often exposed to molecular stress induced by exposure to genotoxic insults, which may include stimuli that cause genomic instability in divergent cellular locations [3]. Dysregulated mitosis may also end up in alteration of optimal telomere distribution due to the strain on the replicating telomerase activity [1–3].

When telomeres shorten below the desired optimal length, the telomere ends remain bare and initiate DNA damage responses—prolonging genomic instability and finally resulting in apoptosis [4]. Thus, assaying telomere length can serve as a biomarker for diverse tumors, including those involving solid organs like the liver [5–7].

In the present study, we used a small series of highly defined patients, where we had matched timed peripheral blood samples (PBS), as well as paired liver biopsies obtained during collection of blood samples from patients with diagnosed hepatocellular carcinoma (HCC) and compared the correlation between the changes of telomere lengths in these defined samples. HCC was diagnosed in patients who had both pre-existing hepatitis B infection and hepatitis C infection or had a non-infectious etiology of HCC. All the documented patients had co-existent advanced cirrhosis confirmed by imaging and liver histopathology. The rationale for the present study was to determine the correlation and reliability of measurement of telomere length as a biomarker for assaying disease persistence and progression in HCC. The main novelty of the present study is matching the correlation of the telomere length alteration observed in cells in the peripheral blood and correlation with the liver biopsy samples.

## Materials and methods

### Patients, sample collection, and ethical considerations

All patients and control subjects enrolled in the present study signed an informed consent form. The study was performed in strict accordance to Helsinki Declaration guidelines and was formally approved by the Institutional Review Board at the Medical School of Nanjing University.

To test the interaction between peripheral blood leukocytes (PBLs) and liver telomere length and the disease persistence and progression of HCC, the present prospective case–control study was designed to compare the telomere with single-copy gene reference standard ratio (T/S ratio) measured by quantitative PCR from HCC patients with or without pre-existing hepatitis B virus (HBV) and hepatitis C virus (HCV) infection to cancer-free controls, with neutralization of variables including sex, race, and alcohol use.

Twenty five patients with pre-existing HBV infection (viral load range was 10^4^−10^6^ copies/ml, 19 patients with pre-existing HCV infection (viral load range was 4 × 10^5^−7 × 10^6^ EQ/ml) and 15 patients with HCC without a prior HBV or HCV infection were used in the present study. All of these patients had confirmed diagnosis of HCC (co-existent advanced cirrhosis confirmed by imaging and liver histopathology) and were at a stage of treatment resistance due to the advanced grades. Fifty subjects with non-malignant diseases that came to the liver clinic or gastroenterology clinic were used for control comparative subjects. Two different time-points were examined: samples were obtained once at diagnosis and after 4 months. This allowed for assessment of disease progression and the reliability of the biomarker assessment. Liver biopsies and peripheral blood were obtained for the study.

### Telomere assessment

Genomic DNA was purified from PBLs collected with the aid of Oragene DNA Kit (DNA Genotek, Kanata, Ontario, Canada) following the manufacturer’s instructions. Liver cancer samples were homogenized using a Brinkmann homogenizer and total cellular DNA was obtained using phenol–chloroform. DNA quantitation was performed by Quant-iT PicoGreen dsDNA Assay Kit (cat. no. P7589; Life Technologies, Shanghai, China). Electrophoresis was performed on 0.8% agarose gels to assess the nucleic acid integrity. DNA samples were refrigerated and stored at −80°C until further analyses. Degraded samples were not used in any part of the study. Same quantities of DNA were loaded for the PCR reactions described below.

Telomere length assay was performed as described previously [8]. The PCR cycle with regards to T (telomeric) PCR consisted of: denaturation at 96°C for 1 min; followed by annealing and extension at 55°C for 1 min. S (single-copy gene) PCR cycle was as follows: denature at 100°C for 1 min, further denature at 95°C for 15 s, annealing at 60°C for 1 s, extension at 72°C for 20 s, 8 cycles, followed by an immediate denaturation at 96°C for another second, annealing at 58°C for a further second, extension at 72°C for 20 s, and repeated at 35 cycles.

The primer sequences for the telomere PCR were *TEL1B* (5′-CGGTTT(GTTTGG)_5_GTT-3′) (final concentration of 100 nm) and *TEL2B* (5′-GGCTTG(CCTTAC)_5_CCT-3′) (final concentration of 900 nm). The single-copy gene (human β-globin) PCR primers were *HBG1* (5′-GCTTCTGACACAACTGTGTTCACTAGC-3′) (300 nM) and *HGB2* (5′-CACCAACTTCATCCACGTTCACC-3′) (700 nm). The composition of the final reaction mixture was composed of 1% DMSO, 20 mm Tris/HCl (pH 8.4), 50 mm KCl, 0.4× SYBR Green I, 200 μm each dNTP, 22 ng *Escherichia coli* DNA per reaction, 0.4 U of Platinum *Taq* DNA polymerase (Life Technologies, Shanghai, China) per 10 μl reaction, with 7 ng of genomic DNA. Reference DNA was always used for standardization of comparisons. The quantitation was made with reference to the control DNA samples that were loaded. All samples were always kept on ice and analyzed using the ABI 7900HT instrument (Applied Biosystems, Life Technologies, Shanghai, China) for analysis.

Inter-assay variability was carefully accounted by running numerous samples of the DNA and thereafter normalized to obtain the final T/S ratio. Inter-run variability was always <5%. If the coefficient of variance was larger, then the entire sample was rerun.

### Statistical analyses

Outliers were not identified in the computed T/S ratios. Data were expressed as mean ± S.E.M. For multiple comparisons, ANOVA was utilized. Between samples, comparison was made by Tukey’s HSD (Honestly Significant Difference) test. A *P*<0.05 was considered to be statistically significant. Correlation analyses was performed to match data distribution between the different sites (blood compared with liver) analysed.

## Results

Datasets were compared by ANOVA. In addition, paired samples were compared by Tukey’s HSD test of unpaired samples to see significance of difference between mean of an individual condition that progressed to HCC (e.g. HCC without HBV or HCV infection compared with controls, HCC in HBV pre-infection compared with controls, and HCC in HCV pre-infection compared with controls).

**Figure 1:**
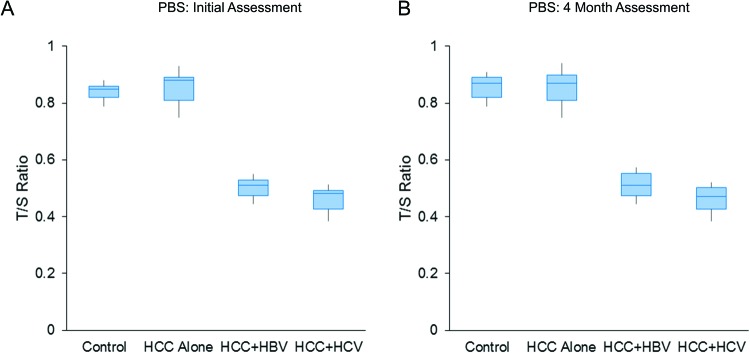
Telomere (T/S ratio) shortening in PBS at diagnosis (A) and after 4 months (B) of patients with HCC, who had either pre-existing serologically confirmed either HBV or HCV infection compared with HCC patients without pre-existing viral infection (HCC alone) or control subjects (*P*<0.001, ANOVA) Patients did not receive any treatment (excluding supportive and palliative care) in the intervening period. Note that similar trend in telomere shortening persisted for 4 months.

In paired samples, telomere shortening was observed in both sets of patients with HCC, who had either pre-existing serologically confirmed either hepatitis B or hepatitis C infection, but not in HCC patients without pre-existing HBV or HCV infection (mean ± S.E.M., 0.82 ± 0.02 (control), 0.81 ± 0.01 (HCC without HBV or HCV infection), 0.47 ± 0.01 (HCC + HBV), and 0.43 ± 0.01 (HCC_HCV), SS3.15, MS 1.58, F 195.29, *P*<0.001, ANOVA; between groups comparison, M1 or M4 compared with M2 and M1 or M4 compared with M3, but not M1 compared with M4, were highly significant, *P*<0.01, Tukey’s HSD test) (Figure 1A).

Importantly, these telomere shortening persisted in PBS obtained after 4 months of the initial assay (mean ± S.E.M., 0.84 ± 0.01 (control), 0.5 ± 0.01 (HCC + HBV), 0.38 ± 0.01 (HCC + HCV), and 0.84 ± 0.01 (HCC without HBV or HCV infection), SS3.82, MS 1.91, F 204.23, *P*<0.001, correlated samples ANOVA; between groups comparison, M1 compared with M2 and M1 compared with M3, but not M1 compared with M4 were highly significant, *P*<0.01, Tukey’s HSD test) (Figure 1B). Because of wide-spread metastasis, as well as diffuse local disease, none of these patients had access to any tumor-specific therapy within these 4 months. They only received supportive and palliative care.

**Figure 2:**
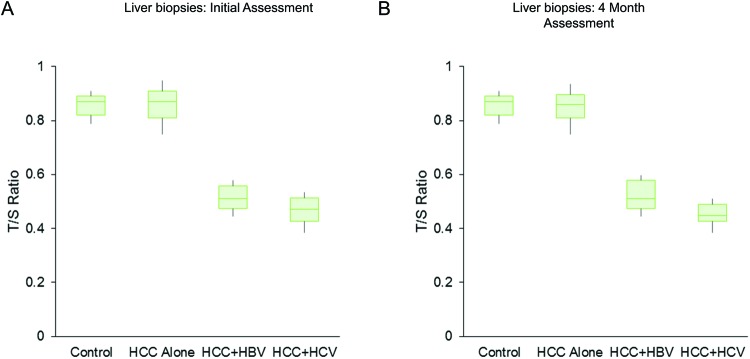
Telomere (T/S ratio) shortening in liver biopsies at diagnosis (A) and after 4 months (B) of patients with HCC, who had either pre-existing serologically confirmed either HBV or HCV infection compared with HCC patients without pre-existing viral infection (HCC alone) or control subjects (*P*<0.001, ANOVA) Patients did not receive any treatment (excluding supportive and palliative care) in the intervening period. Note that similar trend in telomere shortening persisted for 4 months.

The same trend of telomere shortening was also seen in the genomic DNA examined from the liver cancer samples with pre-existing HBV or HCV infection in comparison with control liver samples or HCC samples without any pre-existing viral infection (mean ± S.E.M., 0.84 ± 0.02 (control), 0.86 ± 0.04 (HCC without viral infection), 0.48 ± 0.01 (HCC_HBV), 0.42 ± 0.01 (HCC + HCV), SS3.42, MS 1.72, F 193.12, *P*<0.001, correlated samples ANOVA; between groups comparison, M1 or M4 compared with M2 and M1 or M4 compared with M3, but not M1 compared with M4, were highly significant, *P*<0.01, Tukey’s HSD test) (Figure 2A). Samples from repeat biopsies obtained after 4 months showed the same trend (mean ± S.E.M., 0.84 ± 0.01 (control), 0.85 ± 0.01 (HCC without viral infection), 0.48 ± 0.01 (HCC + HBV), 0.42 ± 0.01 (HCC + HCV), SS3.51, MS 1.76, F 297.63, *P*<0.001, correlated samples ANOVA; between groups comparison, M1 or M4 compared with M2 and M1 or M4 compared with M3, but not M1 compared with M4, were highly significant, *P*<0.01, Tukey’s HSD test) (Figure 2B).

**Figure 3:**
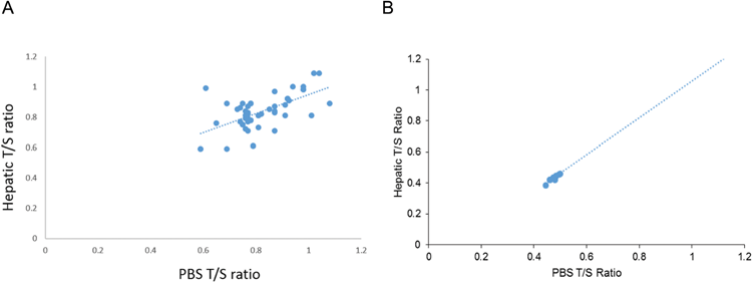
High correlation of telomere lengths between PBS and liver biopsies in healthy subjects (A) or HCC patients with pre-existing HBV infection (B)

The values of telomere ratios matched and closely correlated between PBS and solid organ biopsies of the liver in the control (correlation = 0.63) or HCC patients with pre-existing HBV infection (correlation = 0.93) (Figure 3A and B). None of the datasets were skewed and thus there was no requirement for logarithmic transformation of the data prior to comparison between mean by ANOVA. However, these data merit slight cautious interpretation because of the relatively small sample size used in the present study.

## Discussion

The results of the present study provide the first convincing evidence that PBS may be utilized to assay telomere shortening as a predictor for disease persistence in HCC resulting after HBV and HCV infection, but not in HCC cases without a prior incidence of HBV or HCV infection. Though isolated previous studies have been performed with either blood or liver samples and have suggested that HCC pathogenesis might be related to telomere shortening [9–15], this is the first study in which paired samples have been assayed. It has been previously shown that that leukocyte relative telomere length is an independent prognostic marker for HCC patients treated with transarterial chemoembolization [16]. Given our results, it is likely that circulating viral genome affects the telomerase activity in peripheral blood cells, and thus PBS telomere length assay is a reliable surrogate for disease persistence and progression in the cirrhotic nodules in the liver. Reduction in telomere length limits the cell division capacity of human cells, restrains the regeneration of organ systems during chronic diseases and ageing and also induces chromosomal instability (CIN) as well as initiation of cancer. Previous studies have demonstrated that telomeres are often significantly shorter in tumor tissue, including HCC, compared with the surrounding tissue [17].

CIN results in aneuploidy and chromosomal aberrations in human HCC. Telomere shortening may contribute to this development of CIN. The results of the present study provide incipient opportunity to develop telomere length assessment as a clinical tool for prediction of cancer development, as well as responses to chemotherapy and other treatment modalities. It may be difficult to obtain paired samples all the times. But, because of the ease of obtaining PBS, it is easy to incorporate these studies on a larger scale to assess the validity of the findings of the present study.

## Abbreviations

CIN: chromosomal instability
dNTP: deoxy-ribonucleoside triphosphate
HBV: hepatitis B virus
HCC: hepatocellular carcinoma
HCV: hepatitis C virus
HSD: honestly significant difference
PBL: peripheral blood leukocyte
PBS: peripheral blood samples
S: single-copy gene
T: telomere signal
T/S ratio: standard ratio
